# T-Bet Is Dependent on Stat-4 Inhibiting Acute Colitis but Not Stat-1 Using L4 Somatic Antigen of* Heligmosomoides polygyrus*

**DOI:** 10.1155/2018/8571920

**Published:** 2018-06-07

**Authors:** Agustina Tri Endharti, Sofy Permana

**Affiliations:** ^1^Department of Parasitology, Faculty of Medicine, Brawijaya University, Indonesia; ^2^Biomedical Central Laboratory, Faculty of Medicine, Brawijaya University, Indonesia; ^3^Department of Biology, Faculty of Mathematics and Natural Sciences, Brawijaya University, Indonesia

## Abstract

Helminths may alter the immunoinflammatory reactions of colitis. Proteins derived from* H. polygyrus *have prospective therapy for colitis. The goal of this study was to interpret the protective mechanisms of L4 somatic antigen (LSA) from* Heligmosomoides polygyrus* against an inflammatory response to the pathogenesis of DNBS-induced colitis. Colitis was actuated in mice by rectal instillation of DNBS. The mice were randomly divided into five groups containing control, DNBS alone, and three groups, with different doses of LSA (50, 100, and 200 *μ*g/mL), respectively. Mice initiated colitis by rectal administration of DNBS and after that were immunized with LSA for 14 days. Mice treated with LSA inhibited wasting disease compared with DNBS only group.* The percentages* of* cells* producing IFN-*γ* were reduced by LSA treatment. The level of T lymphocytes CD4^+^IFN-*γ*^+^ cells in the LPL was significantly diminished by LSA at both 100 and 200 *μ*g/mL groups (*p*<0.05). The mRNA expression of T-bet was significantly declined in LSA immunized mice, but not ROR*γ*-T mRNA, whereas GATA-3 expression tended to increase. The activation of STAT-4 significantly reduced LSA-treated mice but not STAT-1. It can be concluded that*** T-bet*** is required for optimal production of*** IFN***-*γ* in colitis.

## 1. Introduction

Ulcerative colitis (UC) is an inflammatory bowel disease that causes long-lasting inflammation in the inner most lining of the colon [1, 2]. UC is a chronic immune disorder affecting the gastrointestinal tract. It is a chronic gastrointestinal illness described by inflammatory changes and mucosal tissue damage [3, 4]. Obsessive signs of UC incorporate ulceration of the mucosa, decreasing and loss of the crypts, and infiltration of inflammatory cells [5, 6]. The signs of colitis stimulated by 2,4-dinitrobenzene sulfonic acid (DNBS) in a rodents models are similar to human UC; these incorporate looseness of the bowels, loss of body weight, shortening of the colon, ulceration of the mucosa, and histopathological lesions as in human UC [5–9]. UC is for the most part related to a T helper type 1 (Th1) immune response [8, 9]. Studies have proposed that Th1 cytokines may assume a part in the enhanced inflammation perceived in the intestinal epithelium of patients with UC [8, 10]. Besides, the mucosa of patients diagnosed with Crohn's disease is dominated by Th1 which contains greater part of CD4+ lymphocytes [8, 10]. UC is associated with proinflammatory cytokines responses, e.g., IFN-*γ*, increasing IFN-*γ* production by T cell lymphocytes [10]. Many researchers described that a treatment including infection with live worms decreases the inflammation related to autoimmune diseases, as Crohn's disease [8, 10]. In contrast, the application of live parasites is disadvantageous because, as a side effect of a live worm treatment, the parasite might invade other tissues of the human host [8–12].* H. polygyrus* is regulated by IFN-*γ*-producing CD4 T cells directly correlated with the percentage of T-bet. GATA-3 controls Th2 activity by inducing Th2 cytokine gene expression. Additionally, T-bet and GATA-3 are transcription factors required to promote Th1 and Th2 differentiation [8, 12]. Generation of Th1 cells from naive precursors requires T-bet signaling that leads to activation of STAT-4 [2, 13]. STAT-4 induces expression of T-bet that acts as a key regulator of Th1 cells [13–15].* H. polygyrus* was effectively used to maintain typical conditions in chronic colitis [2, 16]. Allowing for the fact that helminthes may modulate the inflammatory response of colitis, we explored the effect of* H. polygyrus* on mice in an experimental colitis model. In any case, little is thought about the basic mechanisms of the caring effect of somatic antigen derived from L4* H. polygyrus* larvae against an inflammatory response. We are concerned about proving the expression level of transcription factor reflecting inhibition of inflammation during DNBS.

T-bet performs a crucial function in immune response by modulating the some genes involved in the inflammatory responses [16]. Hence, by monitoring the transcription of inflammatory cytokine genes, T-bet plays a central role in regulating the immune responses and inflammation. T-bet is a critical transcription factor that controls differentiation of Th1 cells; it specifically binds to the promoter of the IFN-*γ* gene and activates its transcription [17–19].

Worm therapy has been established recently. The mechanism of human disease of the numerous worm species can be studied using a mouse model of* H. polygyrus* infection [11, 16]. As confirmed by many, live nematode treatment is considered an alternative therapy [18–20]; this way has a little harm, because patients are infected by live nematodes [17–23]. Additionally, the specific mechanisms sustaining the therapeutic effect of gastrointestinal nematodes are not openly understood. Moreover, the pathology related to worm infection requires for the therapy using live worms to be exchanged with nematodes-derived proteins [2, 11, 16]. The excretory-secretory proteins derived from* H. polygyrus *have a potential in the therapy* o*f colitis [24, 25]. A protein based treatment could overcome the disadvantages of usage that relies on living parasites. The goal of this study was to explore the effect of L4 larvae extract (LSA) on clinical indications of UC, including body weight and extent of occult bleeding, in a mouse model. Studies reported that mice with DNBS-induced colitis exhibit characteristics similar to chronic UC in human [17, 24, 25]. In addition, prolonged and chronic UC develop to a colorectal cancer. Th1 and Th2 cells are associated with increased expression of Gata-3 and reduced expression of T-bet in memory CD4 T cells. CD4 T cells expressing Gata-3 were significantly increased in* H. polygyrus *induced mice. This study also verifies the assessment of the effect of LSA on the expression of T-bet and GATA-3 in DNBS treated colonic tissue. To verify the role of STATs signaling in the regulation of T-bet in LSA-treated mice, we examined whether the T-bet gene targets were generally expressed as STAT1 and STAT4. Therefore, we explored the possibility that STATs activity could play a major role in modulating the LSA mediated T-bet expression.

It is greatly likely that studies employing the models used in the current study might contribute key information about the pathogenesis of human inflammatory colonic diseases in the future. Certainly, the exact mechanism by which LSA affects T-bet and GATA-3 should be investigated further.

## 2. Materials and Methods

### 2.1. H. polygyrus Establishment

Third-stage infective larvae (L3) of* H. polygyrus *were obtained from Jikei University of Tokyo and maintained at Parasitology Laboratory, Medical Faculty, Brawijaya University, Indonesia. Female Balb/c mice were 8 weeks old at the start of the study. Mice were inoculated by oral gavage with 200 L3 of* H. polygyrus* using a 20-gauge ball-tip feeding tube. Infective, unsheathed L3 of* H. polygyrus *(Parasitology Laboratory Collection no. H120) were obtained from fecal cultures of eggs according to Chavarría et al. [22] with modification. L3 collections were propagated and stored at 4°C until used.

### 2.2. Mice

Female Balb/c mice weighing 25-30g were used. They were obtained from Pusvetma Surabaya. The mice were keep under standard conditions of temperature of 25-27°C, relative humidity (55±5%), and 12h/12h light/dark cycle. Mice were given normal drinking water ad libitum during the experimental periods. All experiments were conducted according to the principles of Guide for the Care and Use of Laboratory Animals in Indonesia and were approved by the Ethical Committee of Brawijaya University, Malang, Indonesia (No. 171-KEP-UB).

### 2.3. Preparation of L4 Somatic Antigen (LSA) From H. polygyrus

Previously, third-stage infective larvae (L3) were administered orally for each mouse with single dose of 200 L3. Mice were sacrificed on day 15 after LSA treatment, and then L4 were collected from mouse intestine. LSA was prepared from L4 of* H. polygyrus* according to Chavarría et al. [22]. Briefly, LSA were collected from 100 L4 which were washed several times in Phosphate Buffer Saline (PBS) supplemented with 100mg/mL penicillin and 100 mg/mL streptomycin. Total L4 were homogenized in a cocktail of extraction buffer (2mM EDTA, 10mM PMSF, and 30mM potassium phosphate in PBS) followed by centrifugation at 10.000 rpm for 15 minutes. The final protein concentration of L4 homogenate was measured by the Bradford technique. Total protein of antigen containing <20 endotoxin units/mg was collected and stored at -30°C until used.

### 2.4. Induction of Experimental Colitis and Injection of LSA

**Figure 1:**
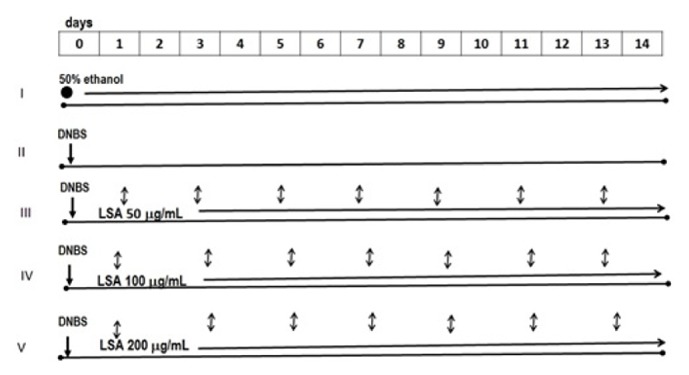
Experimental colitis was induced by administration of DNBS, followed by LSA injection. Firstly, colitis was induced in Balb/c mice by rectal instillation of DNBS followed by regular injection of LSA (L4 somatic antigen) for four times a week (1, 3, 5, 7, 9, 11, and 13). For the experiment, mice were randomly divided into 5 groups: I received 50% ethanol only (control); II was given DNBS only; III–V groups were injected with different concentrations of LSA (50 *μ*g/mL, 100 *μ*g/mL, and 200 *μ*g/mL), respectively, until 14 days. The injection of LSA was started from day 1 until day 13. At the end of 14 days, mice in all groups were sacrificed. ●: 50% ethanol;** ↓**: DNBS; ↕: LSA.

Mice were randomly divided into five groups including group 1 including vehicle (control), groups 2-5 with rectal administration of DNBS, and groups 3-5 that were injected (intraperitoneally) with LSA (50, 100, or 200 *μ*g/mL, respectively) once during 14 days (Figure 1). Colitis was induced by rectal instillation of 5mg/kgBW of DNBS (Sigma Chemical Co., St. Louis, USA) according to procedure described by Morampudi et al. [23] with few modifications. Briefly, colitis was induced in mice by rectal instillation of 5 mg of DNBS/100 *μ*l of 50% ethanol into the rectum through a catheter inserted 4 cm proximally to the anus. The volume of DNBS enema was 100 *μ*L. Thereafter, the mice were kept for 90 seconds in a head-down position to avoid loss of the DNBS. The injection of LSA was started from second day and continued until 14 days. Development of colitis was assessed daily by using an occult blood detection kit (Hemoccult). At the end of day 14, mice in all groups were sacrificed and colon tissues were removed and cleaned and then subjected to histological examination, RNA isolation, PCR, and flow cytometry.

### 2.5. Clinical Assessment of Colitis

Body weight, diarrhea scores, and bleeding scores were assessed daily [17, 24, 25]. Body weight change was monitored. The body weights and occult blood test results were recorded.

### 2.6. Histological Score of Colitis

The microscopic cross sections of the colons were histologically investigated. For histological examination, the colonic tissue was fixed in 10% formalin, dehydrated, paraffin-embedded, processed, and sliced into 5*μ*m thick sections. Furthermore, the sections were deparaffinized with xylene and stained with hematoxylin-eosin. Histological changes were graded semiquantitatively from 0 to 4 according to Erben et al. [26] that described criteria as follows: (0)* no* symptoms of* inflammation* at all; (1) very low levels of leukocyte infiltration and loss of <10% crypts; (2) low level of leukocyte infiltration and loss of crypts between 10% and 20%; (3) high level of leukocyte infiltration, high vascular density and thickening of the colon wall, and loss of crypts between 20% and 30%; (4) severe ulceration, transmural infiltration, loss of goblet cells, high vascular density, and thickening of the colon wall with loss of >30% crypts. To have a quantitative estimation of colon damage, all slides were evaluated using light microscope and scored by two independent observers blinded to the experimental groups.

### 2.7. Isolation and Culture of Mouse Colon Lamina Propria Lymphocyte (LPL) Cells

The culture medium used was RPMI 1640 (Gibco) supplemented with 10 % Fetal Bovine Serum (FBS), 20 mM Hepes, 4 mM glutamine, 100 U/ml penicillin, and 100 U/ml streptomycin (Sigma). Briefly, colon tissues were cut off into 1 mm pieces. This tissue fragments were incubated in HBSS (Sigma) at 37°C under shaking for 30 min in the presence of 200 U/ml type I collagenase (Sigma) and 100 U/ml hyaluronidase (Sigma), 2 mM EDTA, and 25 mM Tris (Sigma). The tissue fragments were suspended and incubated. The incubation procedure was repeated for two times. The supernatant containing the purified epithelial cells was collected by centrifugation at 1200 rpm at 4°C and then separated by Percoll (Sigma) density gradient.

### 2.8. Flow Cytometry and Intracellular Staining

All antibodies used for cell labeling were purchased from eBioscience. For intracellular cytokine measurement, Lamina Propria Lymphocyte (LPL) cells (1x10^6^) were stimulated for 5 h with PMA (1 *μ*g/mL, Sigma Aldrich) and ionomycin (50 *μ*g/mL, BD Biosciences) in the presence of monensin (0.1 mg/mL, Sigma Aldrich) and placed in 37°C and 5% CO_2_. LPL cells were washed with PBS (pH 7.2) and surface-labeled with anti-CD4-FITC (Biolegend, Uithoorn, Netherlands). LPL cells were fixed and permeabilized (Cytofix/Cytoperm, BD Biosciences) and stained intracellularly with anti-IFN-*γ*-PE (BD Biosciences). The stained cells were analyzed using FACS Calibur, and the data were analyzed using Cell Quest Pro software.

### 2.9. RNA Isolation and mRNA Gene Expression

Colon were removed from euthanized mice. The colons were homogenized for total RNA extractions, and oligonucleotide primer was purchased from Integrated DNA technologies (Coralville). Total RNA was isolated using Tri reagent (MP Biomedical, Santa Ana, CA, USA) according to manufacturer's instructions. The yield and purity of RNA were quantified by nanospectrophotometry at 260 and 280 nm. Total RNA (1 *μ*g/ml) was reverse-transcribed to cDNA by using First Strand cDNA Synthesis (Gbioscience) according to the manufacturers. A real time assay was performed according to Light Cycler-Fast Start DNA MasterPLUS SYBR Green I (Roche Applied Science Light Cycler® Diagnostic, USA). Primer sequences were as follows: T-bet forward: 5'-GGATTCTGGGGTTTACTTCTT-3'; T-bet reverse: 5'-TTCTCTGTTTGGCTGGCTGTT-3'; ROR*γ*-T* forward:* 5′-GACAGGGAGCCAAGTTCTCAG-3′; ROR*γ*-T reverse: 5′-TCGGTCAATGGGGCAGTTC-3′; GATA-3* forward:* 5'-ATCAAGCCCAAGCGAAG-3'; GATA-3 reverse: 5'-GCTCTGCCTCTCTAACC-3'; *β*-actin* forward:* 5'-TGGAATCCTGTGGCATCCATGAAAC-3'; *β*-actin reverse: 5'-TAAAACGCAGCTCAGTAACAGTCCG-‘3; STAT-1* forward:* 5'-TCT GTG TCT GAA GTC CAC C-3'; STAT-1 reverse: 5'-CAA GAA AGC GAG CTT AGT GAT AC-3'; STAT-4* forward:* 5′-CAC CTG CCA CAT TGA GTC AAC TA-3′; STAT-4 reverse: 5′-TAA GAC CAC GAC CAA CGT ACG A-3′. The amplification program consisted of the following steps: an initial denaturation at 95°C for 4 min, the 35 cycles of 95°C for 25 seconds of denaturation, 56°C for 10 seconds of annealing, and 72°C for 20 seconds of extension followed by final extension for 3 minutes at 72°C. Each target gene expression was normalized with *β*-actin mRNA content. The relative induction of mRNA expression comparative Ct was calculated using ΔΔCt using the following formula: (Ct target - Ct *β*-actin) treatment - (Ct target - Ct *β*-actin) nontreatment.

### 2.10. Statistical Analysis

All data were presented as the means ± standard deviation. An ANOVA test (analysis of variance) was conducted to determine the statistical significance of difference between groups, with* p* < 0.05 being considered significant.

## 3. Results

### 3.1. Changes in Body Weight following Intrarectal Administration of DNBS

Body growth rate was slightly lower in the mice that received DNBS administration only (Figure 1).

### 3.2. LSA Treatment Attenuates DNBS-Induced Acute Colitis in Mouse Model

**Figure 2:**
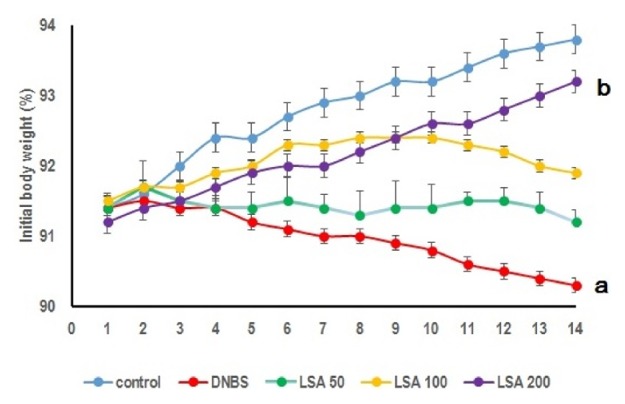
LSA instillation effects on mice with DNBS-induced colitis. Data were obtained from five independent experiments (n = 5), each of which contained five mice (total n = 25, error bar = SD). Body weight loss was observed in mice without LSA instillation (Group II) and Groups III–V were injected with different concentration of LSA. Mice of Group II (DNBS only) showed significant body weight loss compared to LSA-injected mice (a:* p* < 0.05), whereas Group V (LSA 200 *μ*g/mL) injected mice weights were not significantly decreased compared to control. b:* p* < 0.001. Representative results obtained from five independent experiments are shown (ns: not significant).

We examined whether LSA could enhance the DNBS-induced acute colitis. Mice that had been given DNBS developed severe bloody diarrhea followed by an extensive wasting disease. Mice given DNBS alone revealed substantial loss of body weight, i.e., almost 8% between days 4 and 14. The progression of wasting disease was hindered in mice treated with LSA compared with DNBS only group. The injection of LSA clearly enhanced body weight reduction in the recovery period; this effect was continuous during 14 days (Figure 2).

### 3.3. Histological Changes during DNBS-Induced Acute Colitis

**Figure 3:**
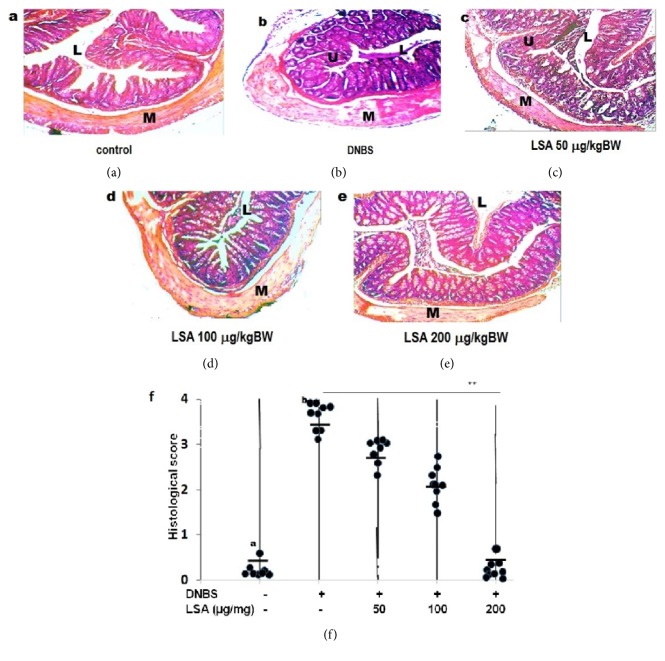
Histological changes with DNBS-induced colitis in mice after 14 days. (a) Mice having instillation of 50% ethanol were used as the control. (b) Mice had rectal instillation of DNBS only. (c–e) Mice had rectal instillation of DNBS and then were injected with different concentration of LSA (50 *μ*g/mL, 100 *μ*g/mL, and 200 *μ*g/mL), respectively. (f) Histological colitis scores of individual mice are shown as circles, and the averages of each group are shown as horizontal bars. Each symbol represents an individual animal. Representative pictures of each group are shown. Groups B and C had significantly higher colitis scores than Group E ((a)* p* > 0.05 and (b)* p* < 0.01, respectively). L: gut lumen; M: muscle; U: ulcer; arrow: inflammatory infiltrate. Magnification 200×.

To assess the effect of LSA on the degree of colitis, mice were sacrificed 14 days after colonic administration of DNBS. The result was consistent with previous findings; DNBS-induced colitis was characterized by the loss of body weight, with extensive ulceration with severe inflammatory cells infiltration in the DNBS only group. The disease was more pronounced in mice only given DNBS. Histopathological analysis revealed a diffuse leukocyte infiltrate in the mucosa of colon sections from DNBS-induced mice (Figure 3(a)). The LSA treatment significantly reduced the extent and severity of the colonic injury, even though injection of 50 *μ*g/mL LSA slightly decreased histological score. These results have demonstrated that LSA is able to reduce colon inflammation, whereas mice given 100 *μ*g/mL LSA had essentially decreased mononuclear cell's infiltration (Figure 3(b)). Further investigation proves that injection of 200 *μ*g/mL LSA was able to reduce inflammatory cell's infiltration. LSA treatment has protecting effects against acute colitis.

### 3.4. Generation of Th1 Cells Requires IFN-*γ* Production

**Figure 4:**
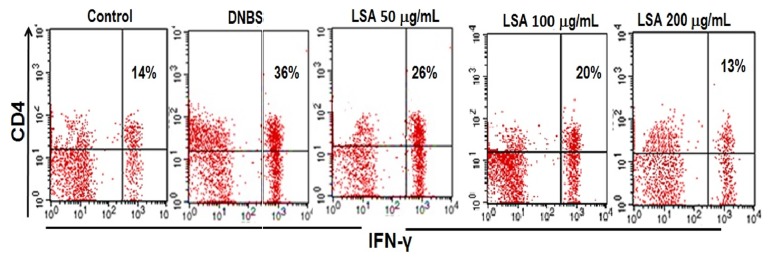
Effect of LSA on suppressing the development of colitis via percentage of IFN-*γ*. A: percentages of lymphocytes T CD4 ^+^IFN-*γ*^+^ were observed in mice with or without LSA injection; B: the percentages of lymphocytes T CD4 ^+^IFN-*γ*^+^ cells were shown. Representative flow cytometry plots show cells gated as CD4^+^IFN-*γ*^+^. Data are representative of four independent experiments with similar results. Percentages of cells in each quadrant are shown inside the panels. Representative results of five mice in each group are shown.

To explore whether LSA could regulate the improvement of Th1, the intracellular cytokines expression in CD4^+^T cell from DNBS-induced mice was characterized by flow cytometry. Colonic injury due to DNBS induction was also considered by an increase in IFN-*γ* production. The percentage of IFN-*γ* from LPL cells was measured by intracellular staining. Our results show that IFN-*γ* production of LPL was significantly higher in DNBS only group, while IFN-*γ* production of CD4^+^ was clearly lower in mice injected with LSA than those in DNBS-induced mice (Figure 4(A)). The percentage of IFN-*γ*^+^CD4^+^ cells in the LPL was significantly declined by LSA treatment at both 100 and 200 *μ*g/mL treatment groups (*p*<0.05) for each group compared to DNBS only group (Figure 4(B)). Results shown are mean±SD, with n=4 replicates in each group, ^*∗*^*p*<0.05, ^*∗∗*^*p*<0.001.

### 3.5. LSA Directly Suppresses Transcription Factor T-Bet DNBS-Induced Th1 Responses

**Figure 5:**
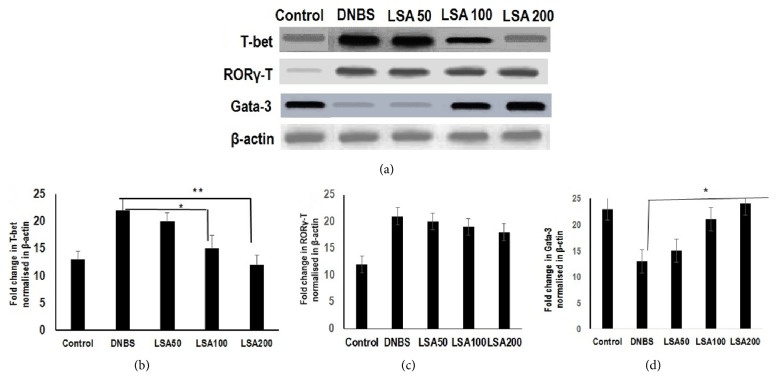
Relative gene expression of T-bet, ROR*γ*-T, and Gata-3 from the colon of CAC models. Amplification was performed by gene-specific primers with 2 *μ*L cDNA for 25 cycles (*β*-actin) or 35 cycles (transcription factor). (b) The fold change in T-bet mRNA (normalized to *β*-actin) expression in colon. (c) The fold change in ROR*γ*−T mRNA (normalized to *β*-actin) expression in colon. (d) The fold change in Gata-3 mRNA (normalized to *β*-actin) expression in colon. Results shown are mean±SD, with n=4 replicates in each group. ^*∗*^*p*<0.05. ^*∗∗*^*p*<0.001 (ns: not significant). Relative gene expression of p53 from the colon of CAC models. RNA was extracted from colons, and p53 gene expression was analyzed by real time PCR and normalized by *β*-actin mRNA. Results are representative of four independent experiments. Data with no statistically significant differences (ns: not significant).

To determine whether T-bet gene targets were generally more highly expressed in Th1 cells than Th2 cells and confirm a functional role of T-bet gene target in LSA-treated mice, we next measured the mRNA gene expression of transcription factor T-bet from LPL cells. Results of real time PCR analysis are shown as bar graphs (Figures 5(a)–5(c)).The result revealed that the mRNA expression of T-bet was significantly diminished in LSA-treated mice (Figure 5(a)), whereas ROR*γ*-T was not significantly altered after treatment of LSA-treated mice (Figure 5(b)). These studies suggest that LSA directly suppresses T-bet in DNBS-induced mice. In contrast, expression of the transcription factor GATA-3 indicated no significant differences between groups (Figure 5(c)).

### 3.6. LSA Treatment Suppressed T-Bet Related Pathway in STATs-Mediated T Lymphocytes Activation

**Figure 6:**
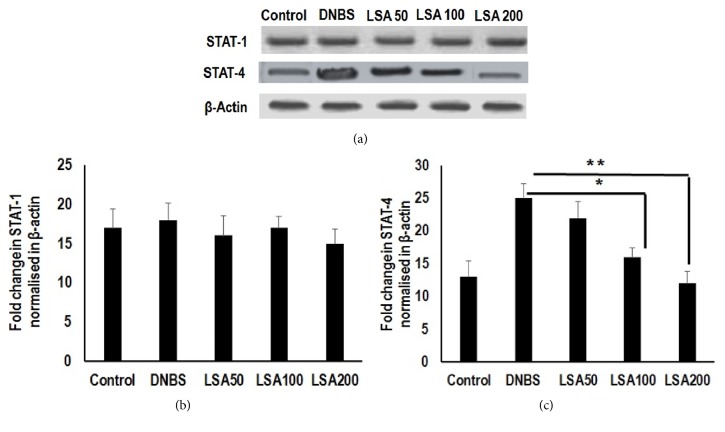
The mRNA expression of STAT-1 and STAT-4. Agarose gel electrophoresis was used for semiquantitative determination of PCR-amplified transcription factor of mRNA in colons; total RNA was extracted and was reverse-transcribed to cDNA. Amplification was performed by gene-specific primers with 2 *μ*L cDNA for 25 cycles (*β*-actin) or 35 cycles (transcription factor). (b) The fold change in STAT-1 mRNA (normalized to *β*-actin) expression in colon. (c) The fold change in STAT-4 mRNA (normalized to *β*-actin) expression in colon. The PCR band density is automatically generated by the ImageJ software. Results shown are mean±SD, with n=4 replicates in each group. ^*∗*^*p*<0.05. ^*∗∗*^*p*<0.001 (ns: not significant).

Moreover, LSA pronouncedly inhibited the transcription expression of genes engaged in the IFN-*γ* associated STATs signaling pathway, including STAT1 and STAT4. STAT-4 expression was significantly reduced in LSA-treated mice. Likewise, STAT-4 expression was significantly higher in mice receiving LSA compared with control counterparts. However, the expression of STAT-1 was not significantly different between DNBS only and LSA-treated groups (Figures 6(a)-6(b)). These findings demonstrate that LSA treatment is able to reduce STAT-4 activation. It was noticed that STAT-4 activity might play a role in regulating LSA mediated T-bet expression.

## 4. Discussion

Helminthic therapy is used to maintain the* balance via modulation *of the* host immune system*. The ability of worms to protect the host from various diseases, such as colitis, encephalitis, rheumatoid arthritis, asthma, and diabetes mellitus has been studied experimentally [23, 24, 26].

It is realized that the inflammatory response is caused by a parasite and the protective consequences are related to the nematode staying in the host's body during treatment. On the other hand, nematodes are allowed to be infected and stay alive, which the patients still find difficult to accept. We think that the somatic antigen derived from nematodes L4 stage induces the immune response to Th1-mediated diseases. The reciprocal cross-regulation between Th1 and Th2 cells suggests that mobilization of a Th2 response by nematodes could reduce the effects of Th1-mediated diseases [24, 26]. In the current study, we also demonstrated that the somatic antigen got from L4 stage* H. polygyrus *has been enabled after DNBS treatment. Further investigation revealed that LSA injection diminished inflammatory cell infiltration into colon. DNBS-induced colitis is mediated by Th1 responses, resulting in enhanced production of T-bet in the inflamed colonic tissue. T-bet assumes a basic part in the pathogenesis of colitis inflammation and critical transcription factor for Th1 cell differentiation. Our experiments demonstrated that LSA injection significantly reduced the percentage of T-bet in LPL cells in DNBS-induced mice. These perceptions indicated that the anti-inflammatory effect of LSA was mediated through a suppressive activity on T lymphocytes. The current study revealed that the LSA has a capacity to suppress colitis induced by DNBS.

Increased DNA-binding activity of T-bet associated with the secretion of high levels of IFN-*γ* has been reported for UC patients [27]. Accordingly, the nuclear localization arrangement of T-bet allowed its translocation into the nucleus, where it binds to DNA and initiates transcription of T-bet dependent genes which were associated with the immune and inflammatory responses. T-bet is recognized as an ideal target for molecular therapies for the treatment of inflammatory diseases [24, 25, 27]. T-bet expression protects against colitis by inducing Th1* expression* on LPL and response of mucosal immune system to intraluminal by regulating IFN-*γ* production in the colon; T-bet also regulates IFN-*γ* production in LPL [25, 27, 28]. These observations suggested that T-bet is induced by STATs signaling during T cell activation.

The aims of the current study were to prove the cure response of DNBS-induced colitis by the L4 somatic antigen. Surprisingly, LSA derived from* H. polygyrus *is able to inhibit Th1 cells. LSA was able to cure colitis in the DNBS-induced mice. Our research has shown that LSA enhances GATA-3 expression. This data indicates that GATA-3 controls Th1 expression. In addition, somatic antigen treatment results in the modulation of immune activity and the reduction of colitis associated with Th1 cells responses [16, 29–32]. These findings indicate a novel function for T-bet as a target gene and provide new perspectives on the pathophysiology of colitis. The anticolitis activity of the somatic antigen attenuates the histopathological changes associated with colitis and declines inflammatory cell infiltration in the mouse colitis model [31–33].

IFN-*γ* has been described as inducing T-bet expression, with a potential to include differentiation of the Th1 cells. Mouse colitis exhibits markedly increased T-bet DNA-binding activity [16, 33]. In addition, LSA could suppress T-bet expression and considerably reduced the production of proinflammatory cytokines, such as IFN-*γ*. Consistently with our results of the current study, LSA treatment suppressed the differentiation of naive CD4 T cells into Th1 subsets which have been considered in the mouse colitis model [16]. In the current study, we observed that the T-bet activity was significantly higher in the colonic tissues of LSA- treated mice than in the control group.

Based on the above results, we propose that LSA inhibits T-bet activity, which leads to the suppression of transcription of inflammatory mediator genes. Our perceptions recommend the existence of an endogenous program for Th2 cell differentiation in vivo, which is usually repressed by T-bet during Th1 cell differentiation. Taken together, these findings suggest that LSA exerts an inhibitory effect on the inflammatory response in colitis through the regulation of T-bet activation. Previous study reported that specific DNA sequences are bound with the transcription factor T-bet that translocates in nucleus and activates gene transcription [16, 34], so by controlling the transcriptional arrangements of inflammatory cytokine genes T-bet plays a critical role for the regulation of immune responses and inflammation, in spite of the fact that molecular mechanisms underlying antiproliferative activity of T-bet remain to be explained.

Interestingly, the activation of STAT-1 did not significantly differ between LSA-treated mice. Importantly, LSA dose-dependently inhibited IFN-*γ* stimulated STAT-4 nuclear translocation in Th1 cells; however it did not influence STAT-1. Despite the suggestion that Stat-4 mainly plays a role in Th1 expansion or survival downstream of T-bet, the role of T-bet in Th1 induction is still controversial [34, 35]. Consequently, the functional correlation between Stat-4 and T-bet in developing Th1 cells may be more complex than is presently appreciated. These data suggest that STAT-4 is associated with a proinflammatory profile, promoting tissue infiltration and proliferation. Therefore, the protein therapies using protein extract are more favorable compared with maintaining nematodes alive in host. We further proved the effect of* H. polygyrus *antigen on DNBS-induced colitis.

## 5. Conclusions

In this study, we found that* LSA injection* had a* significant inhibitory* effect on* mRNA T-bet expression and IFN-γ percentages*. Decreased percentage of intracellular IFN-*γ* producing CD4 T cells in LSA-treated mice caused T-bet downregulation in LSA-treated mice. We demonstrated that injection of LSA may be effective in protecting the mucosa of mice in DNBS-induced colitis, which consequently prevents proinflammatory mediators from acutely increasing. These studies* suggested* that LSA injection could inhibit T-bet differentiation by STAT-4. Our results* suggest* that LSA may has therapeutic potential in colitis. This study indicates that LSA is promising therapeutic agent in ulcerative colitis.

## Data Availability

The data used to support the findings of this study are available from the corresponding author upon request.

## Abbreviations

DNBS: 2,4-Dinitrobenzene sulfonic acid
GATA-3: GATA-binding protein 3
LPL: Lamina propria T lymphocytes
LSA: L4 somatic antigen
STAT: Signal transducer and activator of transcription
ROR*γ*-T: * RAR*-related orphan receptor* gamma*
T-bet: T-box expressed in T cells
UC: Ulcerative colitis.
